# Effectiveness of the Tier 1 Program of Project P.A.T.H.S.: Objective Outcome Evaluation Based on a Randomized Group Trial

**DOI:** 10.1100/tsw.2008.16

**Published:** 2008-01-14

**Authors:** Daniel T. L. Shek, Andrew M. H. Siu, Tak Yan Lee, Chau Kiu Cheung, Raymond Chung

**Affiliations:** ^1^ Centre for Quality of Life, Hong Kong Institute of Asia-Pacific Studies, The Chinese University of Hong Kong, Hong Kong; ^2^ Kiang Wu Nursing College of Macau, Macau; ^3^ Social Welfare Practice and Research Centre, The Chinese University of Hong Kong, Hong Kong; ^4^ Department of Rehabilitation Sciences, The Hong Kong Polytechnic University, Hong Kong; ^5^ Department of Applied Social Studies, City University of Hong Kong, Hong Kong

**Keywords:** positive youth development, objective outcome evaluation, Chinese adolescents

## Abstract

There are two tiers of programs in the Project P.A.T.H.S. (Positive Adolescent Training through Holistic Social Programs). In the Tier 1 Program, teaching units based on different positive youth development constructs are covered. A total of 24 experimental schools (N = 4,121 students) and 24 control schools (N = 3,854 students) were randomly selected to participate in a randomized group trial. Analyses of covariance and linear mixed models controlling for differences between the two groups in terms of pretest scores, personal variables, and random effects of schools showed that participants in the experimental schools had significantly higher positive youth development levels than did participants in the control schools at post-test based on different indicators derived from the Chinese Positive Youth Development Scale. In conjunction with other evaluation findings reported previously, the present study suggests that the Tier 1 Program of P.A.T.H.S. promotes the positive development of Chinese adolescents in Hong Kong.

## INTRODUCTION

There are worrying trends and phenomena related to the development of adolescents in Hong Kong, such as mental health problems, abuse of psychotropic substances, suicide, school violence, and drop in family solidarity[1,2,3]. For helping professionals, one relevant question is how such adolescent developmental problems can be prevented. A survey of literature shows that there are growing efforts to identify at-risk students at an early stage and to develop primary prevention programs that utilize the classroom-based curricular approach[4].

While it is important and meaningful to focus on prevention of adolescent developmental problems, there are several difficulties associated with this approach. First, one may criticize that overemphasis of adolescent developmental problems represents a “pathological” approach in viewing adolescents. Second, in view of the existence of different developmental problems in adolescents, we need a huge number of separate preventive programs (e.g., prevention of bullying, substance abuse, gambling, etc.) and such programs will take up much time if they are implemented in the school context. Third, implementation of preventive programs in the school context would receive strong resistance from the school authority who might argue that their students do not have problems to be prevented. Similar resistance from the parents is also expected. Finally, consistent with the beliefs that “problem free is not fully prepared”[5] and that “young people are not problems to be solved, but resources to be developed”, one may counterargue that it is more useful to consider how adolescents in Hong Kong can develop using a more positive approach. As argued by Shek[6], instead of solely focusing on adolescent developmental problems, it would be helpful to focus our attention on positive youth development programs (i.e., programs that attempt to cultivate the potentials and skills of adolescents).

Unfortunately, a survey of the literature shows that there are very few systematic and multiyear positive youth development programs in Hong Kong, particularly for the junior secondary school students. Although some schools in Hong Kong offer courses on personal development in the names of moral education, civic education, or life education, they lack good coherence and logical continuation among the units and across the levels. In short, school-based youth development programs in Hong Kong usually deal with isolated problems and adolescent development issues only (i.e., deficits-oriented programs). They are also relatively short-term in nature and lack systematic and long-term evaluation.

In view of these concerns and to promote holistic development in adolescents in Hong Kong, the Hong Kong Jockey Club Charities Trust has invited academics of five local universities to form a research team with The Chinese University of Hong Kong being the lead institution and the first author being the principal investigator to develop a multiyear universal positive youth development program in the territory (Project P.A.T.H.S.). There are two tiers of programs in this project. The Tier 1 Program is a universal positive youth development program designed for Secondary 1 to 3 students. There are 10 and 20 h of training for the core program and full program in each school year for each grade, respectively. The Tier 2 Program is specifically designed for students who display greater psychosocial needs at each grade (i.e., selective prevention). The design of the program can be seen in the publications of the project[6,7].

Catalano and associates[8] reviewed the effectiveness of 77 positive youth development programs. Results showed that only 25 programs were successful and different positive youth development constructs were incorporated into the successful programs. These constructs include: promotion of bonding, cultivation of resilience, promotion of social competence, promotion of emotional competence, promotion of cognitive competence, promotion of behavioral competence, promotion of moral competence, cultivation of self-determination, promotion of spirituality, development of self-efficacy, development of a clear and positive identity, promotion of beliefs in the future, provision of recognition for positive behavior, provision of opportunities for prosocial involvement, and fostering prosocial norms. To help adolescents to develop in a holistic manner, these 15 adolescent developmental constructs are covered in P.A.T.H.S., particularly in the Tier 1 Program.

In terms of financial and manpower resources, P.A.T.H.S. can be regarded as a huge project. The Hong Kong Jockey Club Charities Trust has earmarked HK$400 million for the project and more than 220 schools have participated in the project. Obviously, one basic question that should be asked is whether the program is helpful to the students. This question is important because not all positive youth development programs are effective. In the Western context, the review of Catalano et al.[8] showed that among the 77 programs under review, only roughly one-third of them were effective. In the context of Hong Kong, Shek et al.[9] pointed out that evidence-based social work practice was very weak in Hong Kong. As such, there is a need to accumulate research findings on the effectiveness of psychosocial intervention programs. Against this background, it is obvious that rigorous evaluation of P.A.T.H.S. is indispensable.

To provide a comprehensive and complete picture regarding the effectiveness of the project, several evaluation strategies, including objective outcome evaluation, subjective outcome evaluation, qualitative evaluation based on focus groups, student diaries and in-depth interviews, process evaluation, and interim evaluation are employed. Based on the data collected in the experimental implementation phase and full implementation phase, there are results demonstrating the effectiveness of the program via objective outcome evaluation[10], subjective outcome evaluation[11,12,13,14], process evaluation[15], interim evaluation[16], qualitative evaluation[17], and convergence of subjective outcome evaluation and objective outcome evaluation findings[18,19]. Although the above mechanisms provided evidence supporting the positive program effect and Shek[10] reported positive findings based on a one-group, pre-/post-test design, it is noteworthy that objective outcome evaluation based on experimental designs can yield additional important information on the effectiveness of the program. Against this background, this paper reports findings on the effectiveness of the Tier 1 Program of P.A.T.H.S. over a period of 1 year utilizing a randomized, group trial approach.

## METHODS

### Participants and Procedures

Among the 227 schools joining the full implementation phase, there were 132 schools adopting the full program (i.e., 20-h program involving 40 units) and 95 schools adopting the core program (i.e., 10-h program involving 20 units). With the help of the Education and Manpower Bureau (which was renamed as the Education Bureau in July 1, 2007), it was planned that 24 pairs of schools stratified by district with equivalent school characteristics, including district, banding (i.e., academic characteristics of the students), religion, and gender of the students, would be randomly selected from the participating schools adopting the 20-h mode.

Reserve lists of schools were created for each pair of schools in the 24 pairs of schools drawn from the participating schools. For the potential experimental group schools, 31 participating schools were contacted; 24 schools accepted the invitation and 7 schools declined the invitation. For the potential control group schools, 36 participating schools were contacted; 20 schools accepted the invitation and 16 schools rejected the invitation. The rejection rate was high because the schools were eager to join the project without delay for 1 year. As the reserve control school lists were exhausted for four pairs of schools, four equivalent schools not joining P.A.T.H.S. were randomly selected and invited to join the control group by the Education and Manpower Bureau.

Eventually, 19 matched pairs of schools with equivalent background characteristics (with three schools not joining P.A.T.H.S.), four matched pairs with similar background characteristics (with one school not joining P.A.T.H.S.), and one pair of schools that could not be matched were included in the study. For the control schools joining P.A.T.H.S., the students admitted to Secondary 1 in 2006 did not join P.A.T.H.S. in their secondary school years. However, the schools concerned could offer youth enhancement programs that were normally offered to these students. The design of the study is presented in Table 1. The number of students joining the pre- and post-test in the experimental group and control group can be seen in Table 2.

### Procedures

At pre- and post-test, the purpose of the study was mentioned, and the confidentiality of the data collected was repeatedly emphasized to all of the students in attendance on the day of testing. Parental and student consent had been obtained prior to data collection. All participants responded to all scales in the questionnaire in a self-administration format. Adequate time was provided for the participants to complete the questionnaire. A trained research assistant was present throughout the administration process.

**TABLE 1**
**Characteristics of the Experimental Schools (N = 24) and Control Schools (N = 24)**

| Pair | Experimental Group | Control Group | Characteristics |
|---|---|---|---|
| Pair 1 | 1A | 1B | 19 pairs of schools with identical school banding. In each pair, one school was randomly assigned to the experimental group and one school was randomly assigned to the control group. |
| Pair 2 | 2A | 2B | |
| Pair 3 | 3A | 3B | |
| Pair 4 | 4A | 4B | |
| Pair 5 | 5A | 5B | |
| Pair 6 | 6A | 6B | |
| Pair 7 | 7A | 7B | |
| Pair 8 | 8A | 8B | |
| Pair 9 | 9A | 9B | |
| Pair 10 | 10A | 10B | |
| Pair 11 | 11A | 11B | |
| Pair 12 | 12A | 12B | |
| Pair 13 | 13A | 13B | |
| Pair 14 | 14A | 14B | |
| Pair 15 | 15A | 15B | |
| Pair 16 | 16A | 16B | |
| Pair 17 | 17A | 17B | |
| Pair 18 | 18A | 18B | |
| Pair 19 | 19A | 19B | |
| Pair 20 | 20A | 20B | Four pairs of schools with similar banding and randomly assigned. |
| Pair 21 | 21A | 21B | |
| Pair 22 | 22A | 22B | |
| Pair 23 | 23A | 23B | |
| Pair 24 | 24A | N/A | One pair of schools with similar banding, but not randomly assigned. |
| Pair 24 | N/A | 24B | |

**TABLE 2**
**Differences between the Two Groups in Terms of Personal Characteristics**

| Cases | Experimental Group | Control Group | Total |
|---|---|---|---|
| Pretest questionnaire collected | 4,121 | 3,854 | 7,975 |
| Pretest questionnaire available for analysis | 3,792 | 4,044 | 7,863 |
| Post-test questionnaire collected | 3,915 | 3,769 | 7,684 |
| Post-test questionnaire available for analysis | 3,886 | 3,722 | 7,608 |
| Successfully matched cases based on the unique identifier for each participant | 3,298 (49.5%) | 3,358 (50.5%) | 6,656 |

## Instruments

At pre- and post-test, the participants were invited to respond to a questionnaire containing measures of positive youth development, including the Chinese Positive Youth Development Scale (CPYDS). Based on reliability analyses of both pre- and post-test data in the present study, there were some modifications in the composition of the items of the 15 subscales of the CPYDS as follows:

1. Bonding Subscale (six items): $\alpha$ = 0.83 and 0.85 at pre- and post-test
2. Resilience Subscale (six items): $\alpha$ = 0.82 and 0.86 at pre- and post-test
3. Social Competence Subscale (seven items): $\alpha$ = 0.83 and 0.86 at pre- and post-test
4. Emotional Competence Subscale (six items): $\alpha$ = 0.83 and 0.85 at pre- and post-test
5. Cognitive Competence Subscale (six items): $\alpha$ = 0.84 and 0.86 at pre- and post-test
6. Behavioral Competence Subscale (modified five items): $\alpha$ = 0.76 and 0.80 at pre- and post-test
7. Moral Competence Subscale (six items): $\alpha$ = 0.78 and 0.78 at pre- and post-test
8. Self-Determination Subscale (five items): $\alpha$ = 0.76 and 0.80 at pre- and post-test
9. Self-Efficiency Subscale (modified two items): $\alpha$ = 0.50 and 0.56 at pre- and post-test
10. Beliefs in the Future Subscale (modified three items): $\alpha$ = 0.82 and 0.83 at pre- and post-test
11. Clear and Positive Identity Subscale (seven items): $\alpha$ = 0.84 and 0.85 at pre- and post-test
12. Spirituality Subscale (seven items): $\alpha$ = 0.88 and 0.90 at pre- and post-test
13. Prosocial Involvement Subscale (five items): $\alpha$ = 0.83 and 0.83 at pre- and post-test
14. Prosocial Norms Subscale (five items): $\alpha$ = 0.77 and 0.80 at pre- and post-test
15. Recognition for Positive Behavior Subscale (four items): $\alpha$ = 0.76 and 0.80 at pre- and post-test

Several composite indices based on the above measures were also formed. According to Shek et al.[20], the mean of the total mean score based on all 15 subscales could be used as an overall measure of positive development (Indicator 1: $\alpha$ = 0.94 and 0.93 at pre- and post-test). However, as it can be argued that constructs including spirituality, prosocial norms, prosocial involvement, bonding, and recognition for positive behavior are different from the rest of the scales, a summation of 10 subscales (Indicator 2: $\alpha$ = 0.92 and 0.91 at pre- and post-test) assessing psychosocial competence and strengths could be used (i.e., resilience, social competence, emotional competence, cognitive competence, behavioral competence, moral competence, self-determination, self-efficiency, beliefs about the future, and clear and positive identity). Third, based on factor analysis (the details of which can be obtained from the first author), 45 items based on the first three factors involving 11 domains (social competence, emotional competence, cognitive competence, behavioral competence, moral competence, self-determination, self-efficacy, beliefs about the future, clear and positive identity, prosocial norms, and prosocial involvement) were used (Indicator 3: $\alpha$ = 0.96 and 0.96 at pre- and post-test). Finally, based on conceptual analyses of the items, one item was derived for each domain, which resulted in a 15-item key measure (Indicator 4 $\alpha$ = 0.88 and 0.89 at pre- and post-test). The 15 items in this indicator can be seen in Table 3.

## Data Analytic Strategies

In their discussion of the statistical analyses for change data, Allison et al.[21] pointed out that there were four basic strategies. The first strategy was to examine differences between the experimental group and control group at post-test only. As this strategy did not take into account all information, it was not a recommended approach. The second strategy was to conduct a two-way ANOVA (with group and time as the main effect) and examine the interaction effect between group and time. As this approach was often misinterpreted, it was also not recommended. The third strategy was to look at gain scores. However, as the correlation between pre- and post-test scores seldom equals to 1.0, there would be bias in this analysis. The final recommended strategy is to use analyses of covariance to compare post-test scores of the experimental group and control group after controlling pre-test scores. In this study, the final strategy based on analysis of covariance was used. Furthermore, as students in this study were recruited from schools, it could be argued that variations in the outcome measures across groups may also be due to variations in the school

**TABLE 3**
**Items in the Trimmed Scale Involving 15 Items**

| Domain | Item |
|---|---|
| Bonding | When I need help, I trust that my friend will help me. |
| Resilience | When I face adversity, I remain optimistic. |
| Social competence | I understand the rules and expectations when interacting with others. |
| Emotional competence | When I am angry, I can rationally describe my feelings. |
| Cognitive competence | I know how to find the causes of and solutions to a problem. |
| Behavioral competence | I can face criticisms with an open mind. |
| Moral competence | I have the habit of self-evaluation. |
| Self-determination | I am able to make wise choices. |
| Self-efficacy | I can finish almost everything that I am determined to do. |
| Spirituality | Regarding my life goals, there is no progress vs. total achievement. |
| Beliefs in the future | I have confidence that I will be a useful person when I grow up. |
| Clear and positive identity | I am a person with self-confidence. |
| Prosocial involvement | In this school, classmates are encouraged to have mutual help. |
| Prosocial norms | If I do something wrong, I will feel shameful. |
| Recognition for positive behavior | When I help others, my classmates will recognize my behavior. |

characteristics across groups. As such, there is a need to adjust for the random effect of schools when examining the effect of treatment on the outcome variables[22,23,24].

## RESULTS

Using schools as the units of analysis, results showed that the 24 experimental schools and 24 control schools did not differ in the banding of the schools, districts, religion, gender of the students, and source of funding. For the personal characteristics of the participants, results showed that there were no statistically significant differences between the two groups in their sociodemographic background except age (see Table 4). In short, except that the mean age of the control group was higher than that of the experimental group, the background characteristics of the experimental schools and control schools were highly comparable at pretest.

**TABLE 4**
**Differences between the Two Groups in Terms of Personal Characteristics**

| Sociodemographic Variables | Control Group | Experimental Group | Differences |
|---|---|---|---|
| Age | Mean = 3.31 | Mean = 3.18 | Z = 5.66, $p < 0.05$ |
| Gender ratio | Male = 54.77% | Male = 52.70% | $X^2 = 2.48$, $p > 0.05$ |
| Number of persons in the family | Mean = 4.19 | Mean = 4.24 | Z = –1.47, $p > 0.05$ |
| Marital status of parents | Intact families = 82.16% | Intact families = 82.59% | $X^2 = 2.74$, $p > 0.05$ |
| Family on welfare | Yes = 14.79% | Yes = 15.36% | $X^2 = 0.33$, $p > 0.05$ |
| Textbook allowance | Yes = 40.95% | Yes = 38.68% | $X^2 = 3.03$, $p > 0.05$ |

*Note:* Age groups (age 10 or less = 1, age 11 = 2, age 12 = 3, age 13 = 4, age 14 = 5, age 15 = 6, age 16 = 7, age 17 = 8, age 18 = 9, age 19 = 10, age 20 or above = 11).

For the findings based on analyses of covariance, results in Table 5 showed that there were significant differences between the experimental group and control group participants in terms of the four global indicators. For the linear mixed models, the hypothesized models were significantly better than the intercept models ($p < 0.01$ in all cases). In addition, the findings based on the hypothesized models were generally positive. For the estimates of the fixed effects, the experimental group (reference category with a value of 0) performed better than the control group on CPYDS-15 (estimate = –0.035, $p < 0.06$, one-tailed), CPYDS-10 (estimate = –0.04, $p = 0.05$), CPYDS-11 (estimate = –0.07, $p < 0.005$), and KEY15 (estimate = –0.06, $p < 0.05$). These findings generally showed that the experimental group performed better than the control group in terms of the global positive youth development indicators after controlling for pretest scores and age as well as adjusting for the random effect of schools.

**TABLE 5**
**Differences between the Experimental Group and Control Group Based on the Different Indicators Derived from the CPYDS**

| Global Indicator | Estimated Marginal Mean (Control Group) | Estimated Marginal Mean (Experimental Group) | F Value |
|---|---|---|---|
| Findings Based on Analyses of Covariance | | | |
| CPYDS-15 | 4.347 | 4.382 | 6.66, $p < 0.05$ |
| CPYDS-10 | 4.239 | 4.279 | 8.23, $p < 0.005$ |
| CPYDS-11 | 3.799 | 3.858 | 64.06, $p < 0.0001$ |
| KEY15 | 4.340 | 4.399 | 14.94, $p < 0.0001$ |
| Linear Mixed Model Findings Adjusting for the Random Effect of Schools | | | |
| CPYDS-15 | 4.349 | 4.384 | 2.75, $p < 0.06$ (one-tailed) |
| CPYDS-10 | 4.240 | 4.284 | 4.12, $p = 0.05$ |
| CPYDS-11 | 3.790 | 3.857 | 10.86, $p < 0.005$ |
| KEY15 | 4.340 | 4.402 | 5.77, $p < 0.05$ |

*Note:* CPYDS-15 (Mean of the mean scores in 15 subscales); CPYDS-10 (Mean of the mean scores in 10 subscales); CPYDS-11 (Mean of 45 items based on 11 domains); KEY15 (Mean total score based on 15 key items).

## DISCUSSION

The purpose of this paper is to report research findings on the effectiveness of a positive youth development program (Project P.A.T.H.S.) in Hong Kong. There are several unique features of the study. First, to ensure adequate power of the analyses, a large sample size was used. Second, to average out the intrinsic differences between the experimental group and control group, the schools were randomly assigned to the experimental group and control group. Third, a validated measure (Chinese Positive Youth Development Scale) was used. Fourth, both analyses of covariance and linear mixed models were used to analyze the data. Finally, this is the first-known scientific study adopting a randomized group trial design to evaluate a positive youth development program based on a curricular approach in different Chinese communities. As such, it is a ground-breaking attempt in the Chinese culture.

The findings generally showed that compared with participants in the control group, participants in the experimental schools performed better on the different indicators of positive youth development. Adopting a more liberal perspective, findings based on CPYDS-15 suggest that the experimental subjects performed better than the control subjects on the global indicator comprising the 15 domains of positive youth development assessed by the scale, including bonding, resilience, social competence, emotional competence, cognitive competence, behavioral competence, moral competence, self-determination, selfefficacy, beliefs about the future, spirituality, clear and positive identity, prosocial norms, prosocial involvement, and recognition for positive behavior. Adopting a more conservative perspective, findings based on CPYDS-10 suggest that the experimental subjects displayed higher scores on the global indicator reflecting resilience, social competence, emotional competence, cognitive competence, behavioral competence, moral competence, self-determination, self-efficacy, beliefs about the future, and clear and positive identity.

In conjunction with the previous findings based on objective outcome evaluation, subjective outcome evaluation, qualitative evaluation via focus groups, qualitative evaluation via diaries, process evaluation, and interim evaluation, the existing evaluation findings surrounding the Project P.A.T.H.S. basically suggest that the program is an effective one. This is an encouraging finding, particularly in view of the fact that the implementation period of the program was not long. Nevertheless, despite the positive findings of the study, it is noteworthy that there are several limitations of the study. First, the effect size associated with the significant finding was on the low side. This observation may be due to the fact that the duration of the program was short. Second, as only 1 year was involved in the program, only the short-term effect of the program was revealed. Obviously, it would be exciting to examine the program effect over a longer period of time. Third, although the schools did not differ in their background characteristics (when schools were used as units of analyses) and student characteristics, it would be helpful to examine the differences between the two groups in terms of the other school-related variables. With the collection of such information via the School Background Questionnaire (which was still in progress), further analyses of the findings along this line will be carried out in future.

## ACKNOWLEDGMENTS

The preparation for this paper and the Project P.A.T.H.S. were financially supported by the Hong Kong Jockey Club Charities Trust.
